# The relationship between training and competition load characteristics of female boxers—a case study based on collegiate boxers

**DOI:** 10.3389/fpsyg.2025.1517102

**Published:** 2025-05-05

**Authors:** Qinglou Xu, Zhongjie Wang, Lei Liu, Ruiqiu Mao, Changjin Xi, Guoxin Shi

**Affiliations:** ^1^Department of Physical Education, Zhejiang Guangsha Vocational and Technical University of Construction, Jinhua, Zhejiang, China; ^2^Graduate Department, Shenyang Sport University, Shenyang, Liaoning, China; ^3^Department of Basic Teaching, Nanjing University of Finance & Economics Hongshan College, Nanjing, Jiangsu, Zhenjiang, China

**Keywords:** combat sport, menstruation, physical load, weight loss ratio, session-RPE

## Abstract

**Introduction:**

This study examines the relationships between external and internal loads in female collegiate boxers during training and competition, focusing on variations during menstruation and non-menstruation periods. It also evaluates the effectiveness of different monitoring techniques in these contexts, providing guidance for coaches and athletes in load management.

**Methods:**

Twenty-one healthy female collegiate boxers participated, recording data across 18 training sessions and 6 competitions over six weeks. The metrics collected included session-RPE (sRPE), Banister’s Trimp, Stagno’s Trimp, moving distance, high-intensity moving distance, physical load, and the ratio of body weight loss. Pearson correlation analyses were applied.

**Results:**

Correlation analysis revealed that internal and external loads are consistently positively correlated during training and competition. Apart from the training phase, where the correlation between sRPE and Stagno’s Trimp was not statistically significant (*r* = 0.181, *p* > 0.05), correlations between sRPE and other indicators during both training and competition showed medium to high strength (*r* = 0.426 ~ 0.880, *p* < 0.05). During menstruation, the correlations of all metrics were lower than in non-menstruation periods, except sRPE with Banister’s Trimp.

**Discussion:**

The study highlights that the subjective load sRPE can effectively predict and assess the internal load of female boxers, and is more sensitive to internal loads regardless of menstruation status. However, its application to external loads is limited, and objective measurements are recommended during menstruation.

## Introduction

Boxing, a combat sport characterized by its confrontational nature, presents unique challenges in monitoring athletic loads and evaluating systems. Recent rule changes, which base the determination of winners on an overall advantage, underscore boxing’s confrontational essence and increase the physical and psychological demands on athletes. High-intensity performance is required throughout an entire match by boxers, leading to increased physiological stress and perceived exertion. Consequently, monitoring external and internal loads is essential for optimizing training programs and minimizing injury risks (Soligard et al., 2016). Coaches and performance analysts are constantly seeking methods to monitor athletes’ loads during training and competitions. Their goals include optimizing athletes’ psychological and physiological readiness before competitions, monitoring fatigue, and reducing injury risks during events (Yi et al., 2023). The scientification and multidimensionality of load monitoring reflect a general trend in the evolution of combat sports and demand higher scientific literacy from coaches.

Athletic load encompasses the volume and intensity of exercise (Kellmann, 2002) and can be categorized into external and internal loads (Impellizzeri et al., 2005). Tools for measuring external loads may be generic or sport-specific, while those for internal loads may be objective or subjective. Generic tools, such as GPS devices and accelerometers, are utilized across various sports to measure metrics like distance covered and speed (Buchheit et al., 2010). In contrast, sport-specific tools, such as punch trackers in boxing, are designed to measure unique actions like punch volume and force (Dinu et al., 2020). The selection of tools depends on the research or training objectives, with generic tools providing broad physical activity data and sport-specific tools offering detailed insights into the unique demands of a particular sport. External training loads induce physiological stress, which in turn results in internal training loads (Geurkink et al., 2018). Internal training loads are crucial for assessing training effectiveness (Soligard et al., 2016) and are vital indicators of an athlete’s adaptation to training (Lambert and Borresen, 2010; Scott et al., 2013; Takarada, 2003). A systematic review concluded that subjective measures are more sensitive and consistent in detecting acute and chronic changes in athletes’ responses to loads (Saw et al., 2015). Moreover, athletes may engage in longer, more intense training sessions or perceive the load as much harder than planned, potentially leading to poor adaptation. These training plans often overlook the physiological periods of female athletes. Although earlier studies suggested that female athletes might experience decreased performance and increased fatigue during menstruation (Marynowicz et al., 2020), these studies did not explore the relationships among different load dimensions, thus underscoring the importance of monitoring both external and internal loads. While common indicators such as the RPE are subject to subjective influences, they remain crucial for determining the optimal stimuli for biological adaptation (Booth and Thomason, 1991). Objectively assessing internal training loads continues to be a challenge for coaches and researchers (Borg, 1998; Edwards, 1994), who often use a combination of monitoring methods to evaluate training effectiveness and make necessary adjustments, thereby preventing fatigue and injuries caused by overtraining (Jones et al., 2017).

This study examines the external and internal load relationships of female boxers in training and competition scenarios, exploring load characteristics during and outside menstruation, and analyzing the applicability and specific internal relationships of various monitoring methods in different contexts. These variables provide essential reference data for coaches and support personnel to adjust training content for individuals and teams.

## Methods

### Participants

Due to the specificity of the study group (female university boxers), the sample size was limited by the number of athletes in the school’s boxing program who met the eligibility criteria. The final sample size was determined to be 21 participants, based on those who met the inclusion criteria and were willing to participate in the study. In the field of sports science, particularly in studies focusing on combat sports (Haddad et al., 2014), endurance sports (Alexiou and Coutts, 2008), or team sports (De Dios-Álvarez et al., 2021; Scott et al., 2013), similar sample sizes (ranging from 12 to 21 participants) have been used and have successfully yielded reliable conclusions.

Twenty-one female boxers from the Boxing Department of Shenyang Sport University voluntarily participated in this study (M ± SD, 19.5 ± 1.6 years, 164.9 ± 4.4 cm, 58.4 ± 6.8 kg, 21.1 ± 5.4 BMI). These athletes, who had been engaged in boxing and professional training since high school or earlier, had competed in at least ten official or amateur collegiate boxing matches. Prior to the study, a comprehensive survey was conducted to confirm the absence of sports injuries and to ensure that participants were not engaged in other sports training during the experiment. The experiment’s process and objectives were clearly explained to all participants, who then provided written informed consent (The recruitment period for this study runs from 28/03/2024 to 10/04/2024). This experiment was conducted in compliance with the Declaration of Helsinki, and the Ethics Committee of Shenyang Sport University approved the study (Ethics [2024] No. 12).

All participants were eumenorrheic concerning their MC (length 28.6 ± 2.5 days, menstruation 5.6 ± 0.9 days), did not utilize oral contraceptives or any hormone replacement for a least 6 months (JANSE DE JONGE et al., 2019), were free of neurological and non-communicable diseases, MC disorders (e.g., premenstrual dysphoric disorder or premenstrual syndrome) and physical injuries.

### Procedure

Data collection took place at the boxing training venue of Shenyang Sport University over a 6-week period, encompassing 18 sessions of on-site and strength training along with six simulated matches. Each match consisted of five rounds, each lasting 3 min with 1-min intervals, against opponents of similar weight classes, focusing on technical improvement. The training regimen, designed by the coaching team, scheduled sessions three days a week on non-consecutive days, with simulated competitive matches every Sunday. Data were collected following each training session and match. All activities occurred at the same indoor training facility, conducted by the same coaching team, with a 24-h rest period between successive training sessions.

The standardized procedures for the training courses are as follows: Initially, athletes completed 15 min of warm-up exercises and dynamic stretching. This was followed by 15 min of moderate-intensity punching practice without target contact, and 25 min of combat technique sparring focusing on technical enhancement under an experienced trainer’s supervision. Subsequently, they did 15 min of physical training. All athletes concluded the session with 10 min of relaxed shadowboxing and static stretching.

### Instrumentation

The training and competition process was monitored using the MT-sportsT2 wearable device (Momentum Technology, Beijing), designed to accommodate athletes of varying ages and heights. This device features a portable Beidou dual-satellite system with a GPS sampling rate of 10HZ, a three-axis accelerometer and magnetometer sampled at 100HZ, and a three-axis gyroscope with a sensitivity of 2000 degrees per second. The device comprises a data unit, a photonic heart rate sensor, and MTFA3.5 sports software. Data transmission is enabled through Bluetooth with a minimum range of 10 meters. The device is worn on the upper arm, specifically secured around the bicep. Training data were subsequently analyzed using the MT-SPORTS Data Management Cloud Platform. This technology is similar to other widely used wearable devices in sports science, such as GPS and heart rate monitors, which have been extensively validated for measuring various sports activities, commonly in football (Buchheit et al., 2010).

### Menstrual cycle determination

In this study, participants’ menstrual cycle (MC) statuses were determined through a combination of retrospective control and prospective self-reporting via a smartphone application. Although this method may introduce significant errors in assessing the complete menstrual cycle (menstrual, follicular, ovulatory, and luteal phases), it is feasible for accurate prospective identification of menstruation and early follicular subphases (Allen et al., 2015). Due to its non-invasive nature and low cost, this method is highly practical for field studies and is widely applied in various research contexts (Prado et al., 2020).

Specifically, participants were required to record their menstrual cycle dates and symptoms daily during the study period. Before testing, all participants sent the recorder photos of their smartphone app calendar showing the first and last days of their last three MCs, with updates every three days during the experimental cycles. Additionally, at the start of the study, they completed a menstrual cycle questionnaire, which included questions about the regularity of their cycles, health status, and any symptoms experienced during menstruation, and were encouraged to report any irregularities or uncertainties to the research team.

### Internal load indicators

The study utilized the subjective method sRPE for assessing internal load, complemented by Banister’s Trimp and Stagno’s Trimp as objective methods.

sRPE is derived by multiplying the RPE by the duration of the training or competition (Foster et al., 2001a). The CR-10 Borg scale, modified by Foster et al. was employed as depicted in Table 1. Prior to commencement, staff instructed the athletes on the usage of the RPE scale and related precautions, with initial RPE data collected post-simulation training to ensure proficiency in subsequent experimental applications. According to Foster’s research, RPE feedback is obtained approximately 30 min after training concludes (Bartlett et al., 2017). This delay in assessing training intensity is suggested to mitigate the influence of any particularly tense or easy phases toward the conclusion of training sessions (Foster et al., 2001b), ensuring that subjective fatigue accurately reflects the entire training process and not merely the intensity of activities near the end.

**Table 1:** The 10-scale “Rating of Perceived Exertion Scale” of Foster et al. and the modified “Rating of Perceived Exertion Scale.”

Rating (Points)	The 10-scale “Rating of Perceived Exertion Scale” of Foster et al.	The Modified “Rating of Perceived Exertion Scale”
	Descriptor	How tired do you feel now?
0	Rest	Nothing
1	Very, very easy	A little
2	Easy	
3	Moderate	Moderate
4	Somewhat hard	Somewhat tired
5	Hard	Tired
6		
7	Very hard	Very Tired
8		
9		
10	Maximal	Extremely Tired

Banister’s Trimp is determined by multiplying the average heart rate by the training/competition duration (min). It serves as a method to quantify training load, stemming from the relationship between heart rate and blood lactate, which varies with the intensity and duration of incremental exercise; thus, Banister et al. (1975) proposed the training impulse algorithm.

Stagno’s Trimp (Stagno et al., 2007) segments the session into five heart rate zones based on the percentage of maximum heart rate (HRmax), with each zone assigned a weighting factor:

Zone 1: 65–71% HRmax (weighting factor = 1.25).

Zone 2: 72–78% HRmax (weighting factor = 1.71).

Zone 3: 79–85% HRmax (weighting factor = 2.54).

Zone 4: 86–92% HRmax (weighting factor = 3.61).

Zone 5: 93–100% HRmax (weighting factor = 5.16).

Each interval Trimp = interval duration × interval weighting factor.

Heart rate data were collected during training and competitions using a photonic heart rate sensor integrated into the MT-SportsT2 (Model: T2; Brand: Momentum Technology; Beijing, China) wearable device, recording at a sampling rate of 100 Hz. In previous studies, the device was validated for its reliability through its application on football players (Yaqi, 2023). Data were exported post-training and competition using the associated MT-SPORTS Data Management Cloud Platform software. The maximal heart rate (HRmax) for Stagno’s Trimp calculations was determined using the age-predicted formula: 208–0.7 × age. This formula is widely used in sports science to estimate maximum heart rate when direct measurement (such as via maximal exercise testing) is not feasible, providing a practical and reliable estimate for monitoring training loads (Tanaka et al., 2001).

### External load indicators

Physical load refers to the total amount of acceleration and deceleration activities across three dimensions, measured by the variation in three-axis accelerometer data at each data point during a recording. This metric is recorded by the MT-SportsT2 wearable device worn on the upper arm, and data are exported after each training or competition using the MT-SPORTS Data Management Cloud Platform software. This metric helps quantify the cumulative external mechanical stress on the body during three-dimensional movements. In boxing training and competition, it captures various movements such as footwork, punching, dodging, grappling, and running at different speeds, offering a comprehensive assessment of physical activity volume and intensity. Thus, coaches can tailor the training content based on real-time data and planned exercise parameters. Post-activity, this data allows for an analysis of the athletes’ engagement in training, with higher physical load values indicating greater activity and engagement.

Moving distance denotes the total distance an athlete covers during training or competition, measured by the integrated GPS system in the MT-SportsT2 device, with a GPS sampling rate set at 10 Hz. This serves as a macroscopic measure of load volume (Miao et al., 2020). Prior studies have recognized it as an external load indicator (Harley et al., 2010; Buchheit et al., 2010; Atan et al., 2016). High-intensity running distance is defined as the distance covered when the athlete’s heart rate exceeds 150, with heart rate data collected through the MT-SportsT2 device’s photonic heart rate sensor and corresponding distances calculated based on GPS data.

The calculation of the weight loss ratio is as follows:

$$\mathrm{Weight}\;\mathrm{loss}\;\mathrm{ratio}=\frac{Pre\_\mathrm{training}\;\mathrm{weight}-\mathrm{Post}\_\mathrm{training}\;\mathrm{weight}}{Pre\_\mathrm{training}\;\mathrm{weight}}$$

This metric, initially proposed by Hongyou et al. (2015), was validated among soccer players, showing a moderate correlation (*p* < 0.01, *R* = 0.52). It has been adopted as an evaluation indicator for boxers. The protocol is as follows: Body weight measurements are conducted 5 min before and after each training session or match (between 2 PM and 5 PM) in a private room at a stable temperature (20 ± 5°C). Participants must void their bladders and are weighed in their underwear only. Written informed consent for nude weight measurement has been obtained from all participants. To minimize weight measurement errors due to hydration, the head coach and experimental team pre-instructed the athletes to refrain from hydrating during the session. Measurements are performed by the same researcher using the same calibrated digital scale.

### Statistical analyses

Data were analyzed using SPSS (IBM SPSS Statistics for Windows, version 29.0, IBM Corp, Armonk, NY, USA) software following data organization. The Shapiro–Wilk test assessed data normality and Pearson correlation analyses were utilized. According to Hopkins et al. (2009), a correlation coefficient (*r*) of 0 signifies no correlation, 0.1 a low correlation, 0.3 a moderate correlation, 0.5 a high correlation, 0.7 a very high correlation, and 0.9 an extremely high correlation. Analyses were conducted at a significance level of *α* < 0.05, with confidence intervals established at 95%.

## Results

During the 6-week study, which included 18 training sessions and 6 competitions, four participants missed a total of six training sessions and one competition due to menstrual discomfort or other reasons. The remaining athletes completed all tests, yielding a total of 372 valid training data entries and 125 valid competition data entries, of which 20 were collected during menstruation periods.

Correlation analysis revealed that the three internal load indicators (sRPE, Banister’s Trimp, Stagno’s Trimp) and four external load indicators (moving distance, high-intensity moving distance, physical load, weight loss ratio) were consistently and statistically positively correlated and displayed a medium to high strength correlation (*r* = 0.424 ~ 0.621, *p* < 0.05) (Table 2).

**Table 2:** The correlation between internal and external loads (*n* = 21).

Indicator	moving distance (r [95% CI])	*p*	high-intensity moving distance(r [95% CI])	*p*	physical load(r [95% CI])	*p*	weight loss ratio(r [95% CI])	*p*
sRPE	0.426* (0.006~0.719)	0.048	0.438*(0.020~0.726)	0.042	0.533*(0.144~0.780)	0.011	0.611*(0.255~0.821)	0.003
Stagno’s Trimp	0.621*(0.261~0.837)	0.004	0.481*(0.089~0.716)	0.043	0.613*(0.261~0.823)	0.006	0.471*(0.081~0.703)	0.039
Banister’s Trimp	0.433*(0.018~0.722)	0.041	0.436*(0.019~0.724)	0.046	0.585*(0.191~0.804)	0.012	0.424*(0.004~0.708)	0.047

*p < 0.05.

**Figure 1:**
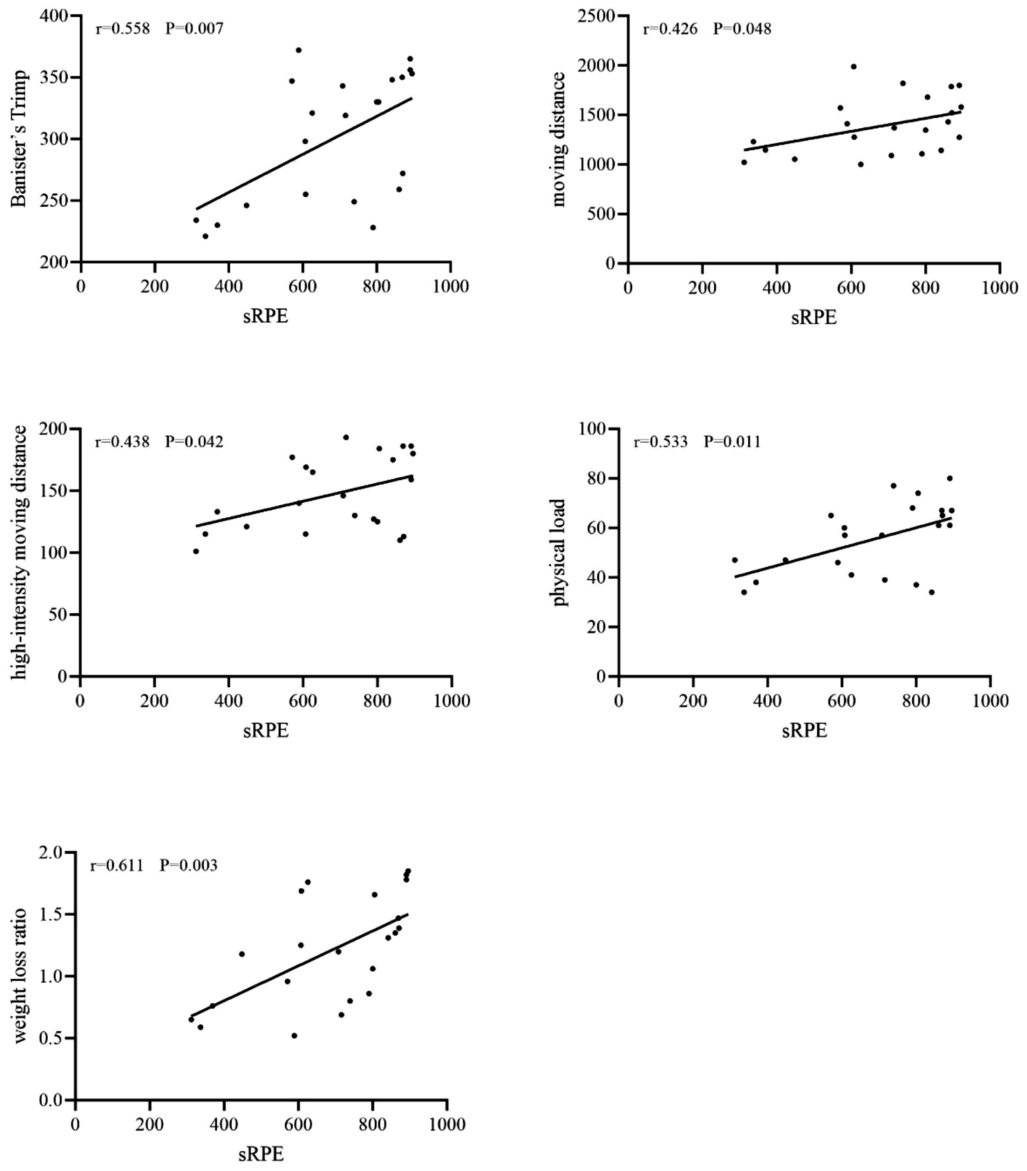
Linear correlation between sRPE and other metrics in training.

**Figure 2:**
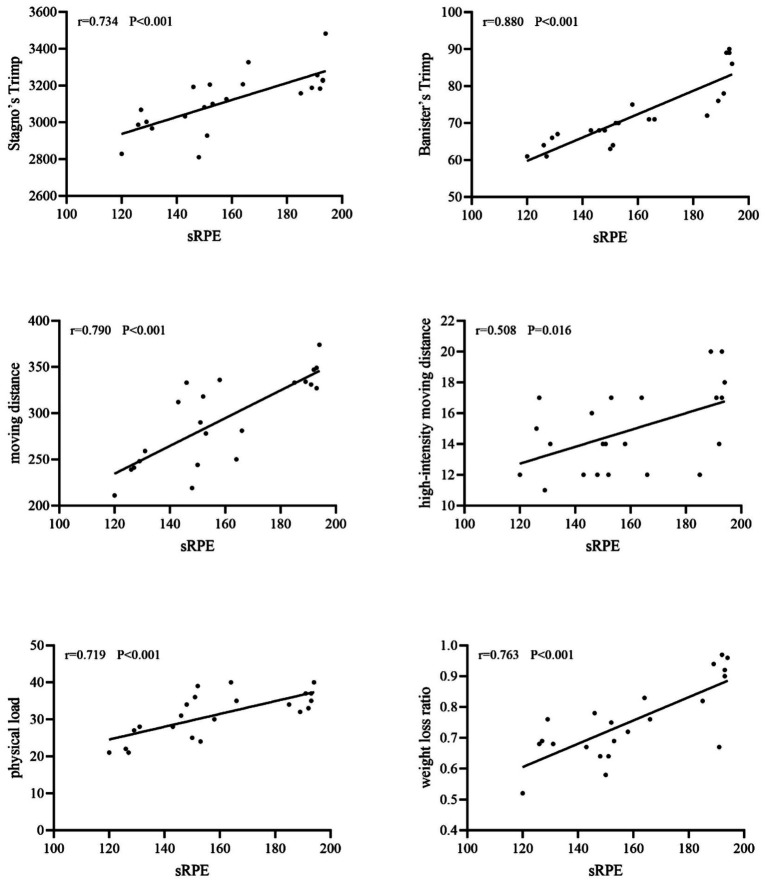
Linear correlation between sRPE and other metrics in competition.

In training scenarios, sRPE was not statistically significant with Stagno’s Trimp (*r* = 0.181, *p* > 0.05) but showed a medium to high strength correlation (*r* = 0.426 ~ 0.611, *p* < 0.05) with other indicators. In competition scenarios, sRPE was highly correlated with two heart rate-based internal loads and four external load indicators (*r* = 0.508 ~ 0.880, *p* < 0.05) (Table 3; Figures 1, 2).

**Table 3:** The correlation between training and competition loads (*n* = 21).

Comparison	Training (r [95% CI])	*p*	Competition (r [95% CI])	*p*
sRPE with Stagno’s Trimp	0.183(-0.258~0.561)	0.415	0.734*(0.452~0.882)	0.000
sRPE with Banister’s Trimp	0.558*(0.178~0.793)	0.007	0.880*(0.728~0.949)	0.000
sRPE with moving distance	0.426*(0.006~0.719)	0.048	0.790*(0.552~0.909)	0.000
sRPE with high-intensity moving distance	0.438*(0.020~0.726)	0.042	0.508*(0.110~0.766)	0.016
sRPE with physical load	0.533*(0.144~0.780)	0.011	0.719*(0.427~0.875)	0.000
sRPE with weight loss ratio	0.611*(0.255~0.821)	0.003	0.763*(0.504~0.896)	0.000

Notes:* *p*<0.05.

For athletes not in their menstrual period, sRPE was highly correlated with all indicators during competition, showing significant results (*r* = 0.551 ~ 0.818, *p* < 0.05). However, during menstruation, correlations of all indicators were lower compared to non-menstrual periods, except sRPE with Banister’s Trimp. Notably, sRPE showed no significant correlations with Stagno’s Trimp (*r* = 316, *p* = 0.347), high-intensity moving distance (*r* = 381, *p* = 0.282), and weight loss ratio (*r* = 402, *p* = 0.241), suggesting diminished reliability of subjective load perception during this phase (Table 4).

**Table 4:** The correlation between during menstruation and non-menstruation periods (*n* = 21).

Comparison	menstruation (r [95% CI])	*p*	non-menstruation (r [95% CI])	*p*
sRPE with Stagno’s Trimp	0.361 (0.128~0.632)	0.347	0.818* (0.687~0.952)	0.000
sRPE with Banister’s Trimp	0.781*(0.478~0.893)	0.000	0.712* (0.448~0.894)	0.000
sRPE with moving distance	0.723*(0.436~0.919)	0.000	0.730* (0.461~0.824)	0.000
sRPE with high-intensity moving distance	0.381 (0.202~0.618)	0.282	0.551* (0.194~0.744)	0.011
sRPE with physical load	0.706*(0.482~0.886)	0.000	0.748* (0.468~0.898)	0.000
sRPE with weight loss ratio	0.402 (0.015~0.691)	0.241	0.713* (0.450~0.897)	0.000

Notes:* means *p*<0.05; data from 20 sessions during menstruation and 125 sessions during non-menstruation periods.

## Discussion

### Training and competition

s-RPE is utilized to assess the internal training load. In this study, we observed that its correlation with Banister’s Trimp and Stagno’s Trimp was significantly higher during competition than in training. This discrepancy can be attributed to the high-intensity intermittent nature of boxing, which contrasts with the more controlled environment of training sessions where athletes typically experience prolonged periods of rest and relaxation. Consequently, athletes spend a greater proportion of time in a high-intensity state during competitions, characterized by briefer rest intervals. This may explain why s-RPE provides a more objective assessment of internal loads under competitive conditions. In line with our findings, Casamichana et al. (2013) reported that sRPE shows a higher correlation with internal load indicators during high-intensity states when compared to traditional heart rate measurements. Alexiou and Coutts (2008) also found that shorter interval times during training correlate more strongly with s-RPE and heart rate indicators in female soccer players. Moreover, research indicates that psychological and educational factors can influence the accuracy of s-RPE in assessing internal training loads (HALL et al., 2005). Given that all participants in this study were from the same university, the variability due to educational backgrounds was minimized. However, the motivation and emotional engagement vary significantly between training and competition settings. Typically, athletes are highly motivated and emotionally charged during competitions, striving to excel and gain recognition from coaches. Conversely, the repetitive and often tedious nature of training exercises tends to dampen motivation and emotional intensity. Therefore, these psychological factors may also contribute to the observed discrepancies in s-RPE assessments of internal training loads.

Another notable observation from our study is the low and statistically insignificant correlation coefficient between sRPE and Stagno’s Trimp under training conditions. We analyze this result using two distinct heart rate-based training impulse algorithms. Research indicates that cumulative fatigue escalates with activity duration, particularly in conditions of high concentration and tension, which results in elevated sRPE post-training (Green et al., 2009), thereby challenging the long-term reliability of sRPE. Conversely, we propose that Stagno’s algorithm, by multiplying different intervals by their respective weighting factors, is more appropriate for high-intensity training. This is because actual training sessions typically comprise substantial low-intensity activities attributable to generally low training loads and limited material. Consequently, in Stagno’s algorithm, more low-intensity intervals are amplified by corresponding weighting factors, thus failing to generate significant high-intensity intervals and resulting in a reduced overall training impulse. Thus, although sRPE demonstrates a higher correlation with Banister’s Trimp in training scenarios, we maintain that Stagno’s Trimp algorithm aligns better with the specific characteristics of boxing. Theoretically, with appropriate training content, it remains a viable indicator for monitoring internal loads (Borresen and Lambert, 2008; Haddad et al., 2014). Nevertheless, care must be taken when utilizing sRPE to monitor internal loads during training sessions characterized by prolonged intervals, low intensity, and sparse density.

In training contexts, the correlation coefficient between sRPE and four external load indicators exhibited low to moderate correlation, while in competition contexts, it exhibited high to very high correlation, similar to the correlation results with two heart rate-based indicators previously mentioned. This finding aligns with the results of Pustina et al. (2017), who identified a very high correlation between metabolic load and sRPE in competitions (*r* = 0.70, *p* < 0.01). Given the causal relationship between external and internal loads, a lower external load generally leads to a reduced internal load. However, individual athletes exhibit varied internal load responses to the same external stimuli. During competitions, compared to training, there is greater physical, physiological, and psychological variability, and factors such as referees, on-site conditions, and opponents’ strength may affect athletes’ subjective fatigue perceptions. Potential psychological factors, such as the motivation to win, anxiety, and increased emotional engagement, tend to be more sensitive to the perception of load and are generally more pronounced in competitive environments than in training settings. Similar to our findings, studies in taekwondo have indicated that perceived exertion is higher during training sessions due to the psychological pressure and intermittent high-intensity nature of the sport (Haddad et al., 2014). In endurance sports like cycling, psychological elements such as motivation and anxiety have been shown to significantly affect perceived exertion during competition, which is consistent with our observations in boxing (Alexiou and Coutts, 2008). In contrast to our findings, Dios-Álvarez et al. reported higher correlations between external load indicators and sRPE during training than in competitions in a study with elite young soccer players (De Dios-Álvarez et al., 2021). Similarly, research by Casamichana et al. (2013) showed a very high correlation between sRPE and running distance during training for semi-professional male athletes (*r* = 0.74, *p* < 0.01). These discrepancies could stem from variations in sample size or training program content, as studies indicate that the accuracy of subjective fatigue perception shifts with proportional increases in time spent in either low or high-intensity training (Borresen and Lambert, 2009). Moreover, our study also examined the relationship between sRPE and high-intensity running distance, revealing only a moderate correlation in training contexts. Echoing our findings, Lovell et al. (2013) found moderate correlations between sRPE and high-intensity movement distance (*r* = 0.62, *p* < 0.01) and physical load (*r* = 0.57, *p* < 0.01) in professional rugby training. However, Scott et al. (2013) observed a very high correlation in training between sRPE and high-intensity running distance (*r* = 0.81). This discrepancy may be linked to the frequency and arrangement of high-intensity training sessions within our boxing team’s schedule, which averaged 90 min and primarily focused on skill enhancement, thus resulting in a lower overall intensity and subsequently a lower correlation coefficient between sRPE and high-intensity running distance. It is noteworthy that physical load in boxing training and competitions involves specific movements such as punching, dodging, and footwork practices. The high correlation of sRPE with these physical loads during competitions can provide specific insights relevant to boxing activities for practitioners without GPS measurement devices.

Furthermore, our study also examined the weight loss ratio, which, to our knowledge, is the first inclusion of this indicator in research on female boxing athletes. Results indicated a high correlation and statistical significance between sRPE and weight loss ratio during both training and competitions (*p* < 0.01). This finding is consistent with research by Hongyou et al. (2015), who assessed pre-and post-training weight (naked) and subjective fatigue in 18 male professional soccer players, demonstrating that the weight loss ratio during training serves as an effective indicator of athletes’ training loads. During physical activities, sweat constitutes the primary component of fluid loss. As exercise duration and load intensity escalate, and under the influence of external environmental factors (the study was conducted during May–June, when weather conditions are relatively hot), elevated body temperature triggers excessive heat dissipation, helping to maintain a moderate thermal balance and ensure effective system coordination. Sweating is the most crucial regulatory mechanism to maintain a relatively constant body temperature during exercise, a process governed by both neural and fluid regulation. The loss of water and electrolytes reduces athletes’ metabolic capacity, leads to the accumulation of metabolic products, and diminishes muscle activity capabilities, resulting in slower movements, reduced accuracy, lack of concentration, and an increased risk of sports injuries (Bu and Liu, 2012). Thus, timely and appropriate replenishment of lost fluids and electrolytes can effectively counteract the adverse effects of heat stress caused by the hot environment and training, thus maintaining and enhancing athletes’ physical capabilities, and potentially eliminating physiological fatigue. Consequently, employing the weight loss ratio as a metric for monitoring training intensity proves meaningful. Finally, it is advisable that several internal and external indicators be combined when assessing loads, which can better guide coaches on the real needs of training sessions and aid in developing training programs to further enhance athletic performance and reduce injury rates.

### Menstrual and non-menstrual periods

This study further investigated the load states of athletes during their menstrual and non-menstrual periods in competitive settings. Results demonstrated that during the menstrual phase, the correlation between sRPE and two heart rate-based objective internal load indicators was significantly lower compared to the non-menstrual state. Additionally, the correlation of sRPE with Stagno’s Trimp during the menstrual period proved to be statistically insignificant. In a related study, Gamberale et al. (1975) examined physiological and psychological changes in 12 regularly menstruating women aged 20 to 34. These participants underwent testing thrice: on the first or second day of menstruation, 10–18 days post-menstruation onset, and 2–6 days prior to the anticipated start of the subsequent menstruation. Each subject engaged in a 6-min cycling task on an ergometer at workloads of 40 and 70% of VO2max. Findings revealed that subjective fatigue perceptions were more pronounced during menstruation than in the periods before or after, at identical heart rates. Conversely, Higgs and Robertson (1981) observed that participants reported heightened subjective fatigue only during high-intensity exercises at 100% VO2max within the menstrual period. This variation may stem from the differing timing of tests within the menstrual cycle. Higgs and Robertson conducted tests on specific days of the menstrual cycle, whereas our study spanned the entire cycle, potentially capturing a broader range of hormonal fluctuations. Research indicates that in female athletes, levels of serum testosterone, maximum oxygen uptake, hemoglobin, and blood cells are ranked as follows: luteal phase > follicular phase > menstrual phase (Han, 2015), suggesting that varying test timings can alter subjective experiences. However, both studies corroborated that subjective fatigue perceptions are more elevated during menstruation than non-menstruation, with multiple investigations confirming that hormonal fluctuations induce significant physiological and psychological changes during menstruation in female athletes (Prado et al., 2020). The heightened sRPE and subjective fatigue perceptions toward external loads during menstruation observed in this study further validate these findings. Moreover, variations in study design, participant characteristics, and exercise protocols might also affect the outcomes. Higgs and Robertson utilized a continuous cycling task, whereas our study involved high-intensity intermittent activities (boxing), and results indicated that the reliability of sRPE was lower during menstruation across various training and competition scenarios. These differences highlight the importance of considering the timing of the menstrual cycle and the type of exercise when interpreting data on perceived exertion.

Furthermore, our study revealed that the correlation between sRPE and high-intensity movement distance and weight loss ratio during menstruation was not statistically significant. However, the correlation between sRPE, movement distance, and physical load was significant and demonstrated a high degree of correlation. Does this result suggest that sRPE is not effective for monitoring internal loads during competitions for athletes in their menstrual period? We propose that this outcome is primarily attributable to two factors: firstly, Stagno’s Trimp, as mentioned previously, is more suited to high-intensity intermittent team sports, thus providing more accurate results that align with the characteristics of the sport. Secondly, the result is influenced by the physiological traits of female athletes during menstruation. In this phase, the corpus luteum regresses, and the concentrations of estrogen and progesterone in the blood significantly decrease, causing symptoms such as irritability, restlessness, insomnia, and headaches. These symptoms affect the psychological perceptions of female athletes. The validity of subjective perceptual assessments under hormonal fluctuations has been examined in several studies. For instance, Elliott-Sale et al. (2020) noted that exercise performance may significantly decline during the early follicular phase of the menstrual cycle compared to other phases. Janse de Jonge et al. (2012) found that the perception of physical exertion is higher during the luteal phase of the menstrual cycle in hot, humid conditions; meanwhile, Oosthuyse and Bosch (2010) reported that menstrual cycle stages affect exercise metabolism and the perception of physical exertion. Consequently, the values reported on subjective fatigue scales during menstruation may be biased, leading to a lack of significant correlation between reported subjective internal loads and objective internal load indicators. Additionally, the body begins to retain water during menstruation (Sawai et al., 2018), resulting in slight weight gain or swelling, thereby reducing fluid loss during physical activity. Despite individual variations, these factors raise concerns about the effectiveness and reliability of sRPE during menstruation. To address this limitation, it is recommended not to use sRPE alone for assessing load during training or competition in menstrual periods. Instead, combining sRPE with objective measurements or employing hormonal monitoring can provide more reliable internal load values, enhancing the accuracy of load monitoring for female athletes during their menstrual cycles. In summary, the psychological and physiological changes during menstruation influence the subjective fatigue perceptions of female athletes. The alterations in hormones and emotions intensify the subjective fatigue experienced during high-intensity activities. Therefore, the heightened subjective fatigue of female athletes during menstruation should be fully acknowledged, and post-competition recovery should be thoroughly addressed.

### Limitations

The study has several limitations, including a relatively small sample size (*n* = 21), individual variations in menstrual cycle characteristics, and the potential influence of external factors such as nutrition, sleep, and hydration. These limitations may affect the generalizability of the findings and highlight the need for future studies with larger sample sizes, detailed menstrual cycle tracking (such as measuring estrogen or progesterone concentrations), and controlled external factors. For instance, future research could utilize standardized psychological questionnaires, such as the MDQ, to quantify the psychological impact of menstruation on subjective fatigue. Additionally, more objective measurement methods, such as HRV and blood biomarkers, could be used to more accurately monitor internal loads during menstruation. Furthermore, advancements in wearable technology can provide real-time physiological data, offering new insights into the effects of menstrual cycle phases on performance and recovery.

### Practical applications

The outcomes of this research present multiple practical implications for athletes, sports coaches, and scientists, especially concerning female boxers in terms of managing their training and competitive demands. Initially, the research emphasizes the necessity of incorporating menstrual cycle considerations into the development of training regimens. Coaches are equipped with the knowledge to tailor strength and stamina training intensities and volumes during menstruation, focusing more on skill development and low-intensity aerobic exercises. This adjustment aids in mitigating the risk of overtraining and injuries while preserving peak performance levels. Moreover, during the follicular phase, when athletes may exhibit elevated energy levels, coaches are encouraged to amplify the vigor of strength and conditioning workouts to leverage enhanced athletic performance. Additionally, the research advocates for the integration of subjective evaluations (such as sRPE) with objective indicators (like Banister’s Trimp, Stagno’s Trimp) for more precise training load assessments, particularly amid hormonal shifts. This method enables coaches to base training adjustments on robust data, optimizing training impact and athlete recuperation. Furthermore, the importance of personalized training schedules that accommodate menstrual cycle fluctuations is highlighted, alongside advanced recovery protocols (including optimal hydration, nutrition, and sleep) during menstruation to alleviate fatigue and uphold performance. Lastly, this investigation lays the groundwork for future innovations in wearable technology and biomarker studies, aiming to refine load monitoring and training program development for female athletes in high-intensity sports such as boxing.

## Conclusion

During training and competition, female boxers exhibit a positive correlation between internal and external loads. The subjective load indicator, sRPE, effectively predicts and assesses internal loads but is less reliable for external loads. The correlation between external load indicators and sRPE values is weaker during training and stronger during competition. sRPE is consistently more sensitive to internal loads, independent of menstrual status; however, during menstruation, it is prudent to depend more on objective measurements and to cautiously utilize Stagno’s Trimp and the weight loss ratio for load assessment.

## Data Availability

The data that support the findings of this study are available from the corresponding author, upon reasonable request.
